# Motor Cortical Activity during Observing a Video of Real Hand Movements versus Computer Graphic Hand Movements: An MEG Study

**DOI:** 10.3390/brainsci11010006

**Published:** 2020-12-23

**Authors:** Yu-Wei Hsieh, Meng-Ta Lee, Yu-Hsuan Lin, Li-Ling Chuang, Chih-Chi Chen, Chia-Hsiung Cheng

**Affiliations:** 1 Department of Occupational Therapy and Graduate Institute of Behavioral Sciences, College of Medicine, Chang Gung University, Taoyuan 33302, Taiwan; mengtalee@gmail.com; 2 Healthy Aging Research Center, Chang Gung University, Taoyuan 33302, Taiwan; 3 Department of Physical Medicine and Rehabilitation, Chang Gung Memorial Hospital, Linkou 33305, Taiwan; lchuang@mail.cgu.edu.tw (L.-L.C.); claudia5477@gmail.com (C.-C.C.); 4 Department of Physical Medicine and Rehabilitation, Cathay General Hospital, Taipei 10630, Taiwan; u58868@hotmail.com; 5 School of Physical Therapy and Graduate Institute of Rehabilitation Science, College of Medicine, Chang Gung University, Taoyuan 33302, Taiwan; 6 School of Medicine, College of Medicine, Chang Gung University, Taoyuan 33302, Taiwan; 7 Department of Psychiatry, Chang Gung Memorial Hospital, Linkou 33305, Taiwan

**Keywords:** action observation, virtual reality, magnetoencephalography, primary motor cortex

## Abstract

Both action observation (AO) and virtual reality (VR) provide visual stimuli to trigger brain activations during the observation of actions. However, the mechanism of observing video movements performed by a person’s real hand versus that performed by a computer graphic hand remains uncertain. We aimed to investigate the differences in observing the video of real versus computer graphic hand movements on primary motor cortex (M1) activation by magnetoencephalography. Twenty healthy adults completed 3 experimental conditions: the resting state, the video of real hand movements (VRH), and the video of computer graphic hand movements (CGH) conditions with the intermittent electrical stimuli simultaneously applied to the median nerve by an electrical stimulator. The beta oscillatory activity (~20 Hz) in the M1 was collected, lower values indicating greater activations. To compare the beta oscillatory activities among the 3 conditions, the Friedman test with Bonferroni correction (*p*-value < 0.017 indicating statistical significance) were used. The beta oscillatory activities of the VRH and CGH conditions were significantly lower than that of the resting state condition. No significant difference in the beta oscillatory activity was found between the VRH and CGH conditions. Observing hand movements in a video performed by a real hand and those by a computer graphic hand evoked comparable M1 activations in healthy adults. This study provides some neuroimaging support for the use of AO and VR in rehabilitation, but no differential activations were found.

## 1. Introduction

Action observation (AO), an emerging new rehabilitation approach, is defined as a dynamic process during which an observer can understand what other people are doing by simulating the actions and the outcomes that possibly follow from the observed motor act [1,2]. During AO, the observers are required to carefully watch movements or daily actions performed by healthy people in video clips as if the observers themselves executed these actions to restore the neural structures normally recruited during the execution of these actions [3]. Human mirror neuron system [4] and AO networks, such as ventral premotor cortex (PMv), dorsal premotor cortex (PMd), primary motor cortex (M1), pre-supplementary motor area (SMA), inferior frontal gyrus (IFG), and inferior parietal lobule (IPL), are activated when individuals learn motor actions via observation, imagery, imitation, and execution [5]. Activation of these brain areas following AO may facilitate the subsequent execution of the movements by directly matching the observed action to the internal simulation of that action. Furthermore, AO could also reinforce corticospinal excitability and reduce intra-cortical inhibition in the M1 [6]. Thus, AO enables individuals with stroke to relearn motor skills via the activations of the internal action-related representations.

Over the last decade, virtual reality (VR) has been found to help stroke patients to improve their motor function by providing simulated real-life objects and events and by generating multi-sensorimotor feedback via computer technology [7]. The visual feedback in VR modulates contralateral or ipsilateral M1 activity in patients with stroke who observe a virtual hand on the same or opposite side while moving their own hand [8,9,10]. VR utilizes the tailored manipulation of visual feedback and simulates real situations to enable individuals to improve motor and daily function.

Observation of actions is critical in motor imitation and in the acquisition of new motor skills [11]. The fundamental linkage between the observation of body movements and the activation of motor responses can be traced to the human infancy [12,13,14], especially for behavioral imitation with evolutionary significance in preverbal infants from a perspective of developmental cognitive neuroscience [13]. AO and VR provide different types of visual stimuli to trigger brain activations and help people learn. During AO, people commonly observe movements performed by another healthy individual in video clips. During VR, people watch computer-mediated movements executed by an animated or a virtual hand in a virtual scene. Regarding the authenticity of these two kinds of visual stimuli, the movements observed in AO are relatively real, whereas those in VR are virtual. The neural mechanisms of observing video movements performed by a healthy person’s real hand versus those performed by a computer graphic hand remain uncertain. This study aimed to investigate by magnetoencephalography (MEG) the differences in the effects on motor cortex activations of observing the movements of real versus computer graphic hands.

## 2. Materials and Methods

### 2.1. Participants

A total of 20 healthy adults (11 females) participated in the present study. Their ages ranged from 42 to 77 years of age (54.7 ± 9.5 years). All participants were right-handed, as identified by self-report and Edinburgh Handedness Inventory. This study was approved by the Institutional Review Board of Taipei Veterans General Hospital (2019-02-002AC). All participants provided written informed consent before commencement of the study.

### 2.2. Experimental Design

**Figure 1:**
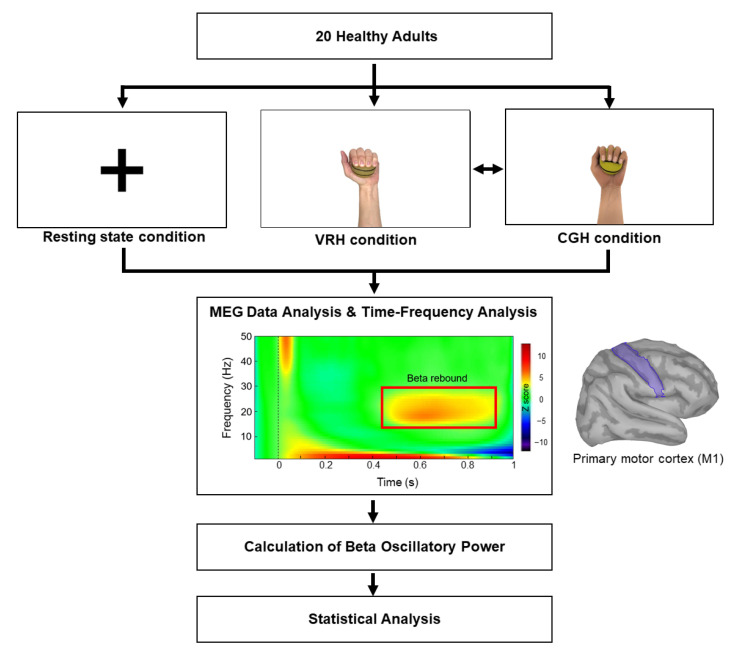
Flowchart of experimental procedure. The beta rebound oscillatory power (red rectangle), ranging from 400 to 900 ms after the onset of electrical stimulation, represents the M1 activity. Notes: CGH = the video of computer graphic hand movements; VRH = the video of real hand movements; M1 = primary motor cortex.

All participants were comfortably seated in an MEG environment and instructed to watch different visual presentations in the three experimental conditions, which were conducted on the same day. Figure 1 shows the study procedure. The 3 experimental conditions included the following: (1) The resting state condition: Each participant was asked to fixate their eyes on a crosshair reticle presented on the screen. (2) The video of real hand movements (VRH condition): Each participant was instructed to remain relaxed and watch a video of a healthy adult gripping and releasing a ball. (3) The video of computer graphic hand movements (CGH condition): Each participant was instructed to watch computer graphic hand movements of gripping and releasing a ball. All conditions continued for 4 to 4.5 min. The videos were consisted of short clips that are looped each 15 s with gripping the ball (10 s) and releasing (5 s), in which 16 to 18 repetitions of gripping and releasing the ball were performed within the 4 to 4.5 min. Except for the resting state condition, the order of the 2 experimental conditions (VRH and CGH) was counterbalanced across the participants.

During all experimental conditions, intermittent electrical stimuli were simultaneously applied to the median nerve of the participant’s left hand with an electrical stimulator (Konstantstrom Stimulator, Schwind, Erlangen, Germany). All pulses consisted of a 0.2 ms constant-current square-wave with a constant interstimulus interval of 1.5 s and a stimulus intensity set 20% above the motor threshold for obtaining cortical responses with a great signal-to-noise ratio [15,16]. The aforementioned stimulus intensity was measured individually for each participant before MEG recordings, and the intensity values were the same among different conditions within the participant. Empirical evidence has indicated that beta oscillations originate in the M1 [17], which could be captured in the right hemisphere of healthy individuals due to electrical stimulation of the left hand in this study. The beta oscillations attenuated in magnitude following median nerve stimulation and then showed a relative increase above the pre-stimulus baseline level within the time window of 0.4 to 0.9 s (i.e., beta rebound) [18,19]. This rhythmic activity would be partially suppressed while participants observed another’s hand movements [15]. Thus, greater suppression of the expression of beta rebound indicated greater activation in the M1 [19], which was considered to be indicative of the functional state of the M1 [20]. Each participant was asked to complete all conditions under instruction and to ignore the electric stimuli during the experimental period.

### 2.3. MEG Recordings

The neuromagnetic data were acquired with a 306-channel MEG (Vectorview, Elekta Neuromag, Helsinki, Finland) in a magnetically shielded room. The 204 planar gradiometers detecting the strongest signals directly above activated cerebral regions were analyzed. The positions of the anatomical fiducial points (i.e., the nasion and the left and right preauricular points), four head position indicator (HPI) coils, and scalp outline were determined with a 3D digitizer. The anatomical fiducial landmarks were used to establish the head coordinate frame. Before each MEG recording, the exact head location relative to the sensor array was computed through HPI coils. Moreover, further ~100 head points uniformly distributed on the head surface were digitized using a 3D digitizer. All MEG signals were acquired at a sampling rate of 1000 Hz with an on-line bandpass of 0.1 to 120 Hz. Single-trial epochs were 1100 ms with a 100 ms pre-stimulus interval for baseline correction. During MEG scanning, the participants were directed to remain in a comfortable sitting position without moving their heads. Short breaks (about 2 min) between different experimental conditions were provided. 

### 2.4. MEG Data Analysis

To correct strong interference resulting from external (i.e., eye blinks or cardiac artifacts) and nearby sources on the MEG data, the temporal signal-space separation method was performed in this study [21]. The mean numbers of trials among the 3 conditions did not significantly differ (resting state = 121.70 ± 42.01, VRH = 115.50 ± 28.16, and CGH = 111.10 ± 10.37; *p* = 0.93). The modeling of M1 beta activity was obtained by Brainstorm software [22]. The MEG data contaminated by prominent electrooculogram artifacts were first removed through the signal-space projection method. An overlapping-sphere method was applied to solve the forward problem of MEG recordings [23]. Next, the cortically constrained source activation was reconstructed by the depth-weighted minimum norm estimate (MNE), with about 15,000 elementary current dipoles assigned over the cortical envelope. The individual’s MNE source map was then rescaled according to the ~100 head points, with the default setting in the Brainstorm software. The MNE source maps from each condition were averaged onto the ICBM152 brain template, on which cortical spatiotemporal dynamics of measured neuromagnetic responses were displayed. An M1 region with the most prominent activation, seen as the region of interest, was manually scouted as a cluster of vertices corresponding to 4–5 cm^2^. 

For characterizing the spectral responses of the identified M1, we used the Morlet wavelet-based time–frequency approach to decompose single-trial evoked potentials from the raw data. The settings of the input parameters included (1) central frequency = 1 Hz; (2) time resolution (full width at the half maximum) = 3 s; (3) time definition = −100 to 1000 ms with 1 ms steps; and (4) frequency definition = 1 to 50 Hz with 1 Hz steps. After transformation, the power of signal fluctuations between 1 and 50 Hz in each participant was shown 1 Hz steps. The beta rebound oscillations ranging from 400 to 900 ms were considered as an indicator of M1 activity (Figure 1). The average strengths of the most robust beta rebound activities (2 Hz for consecutive bins) in the M1 were identified and computed from the mean of 100 ms centering peak latency of beta oscillation (i.e., 50 ms prior to and after the peak) [24]. The time-resolved magnitudes of all elementary dipoles were normalized based on their fluctuations over the pre-stimulus baseline. Subsequently, the z-score values of M1 beta rebound oscillation from all participants were calculated as the mean values in each condition. A smaller beta oscillatory power indicated greater M1 activations.

### 2.5. Statistical Analysis

The nonparametric Friedman test was used to examine the within-group differences of beta oscillatory activities among the 3 experimental conditions, including the resting state, CGH, and VRH conditions. The Wilcoxon signed-rank test with Bonferroni correction was applied for post-hoc comparisons. For the pairwise comparisons, a *p*-value < 0.017 indicated statistical significance.

## 3. Results

**Figure 2:**
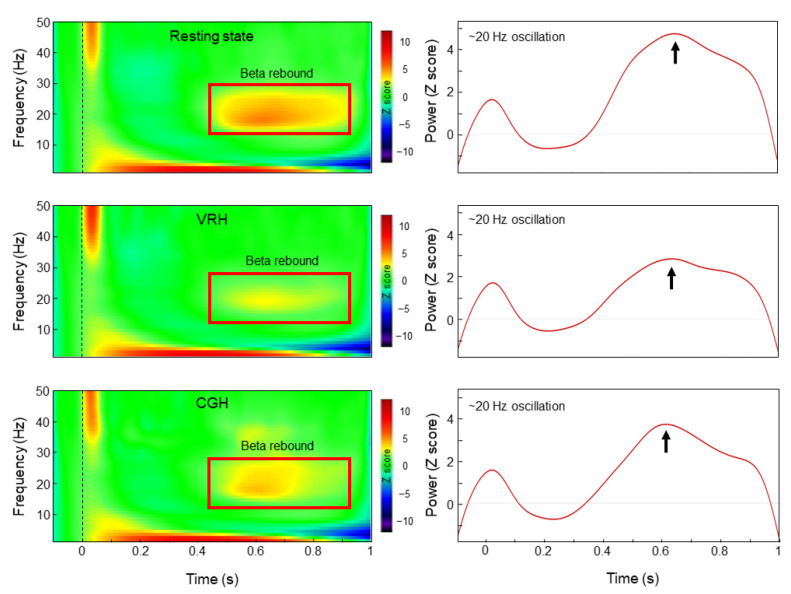
The grand-averaged time–frequency maps and corresponding beta rebound oscillations of M1 in the 3 conditions. The left panel presents time–frequency maps of electricity-induced beta rebound oscillations (red rectangles) averaged across participants in the 3 conditions: resting state, CGH, and VRH. The right panel shows the temporal evolution of the beta oscillations, which were identified as the mean strength of the most reactive frequency bands (2 Hz), relative to the baseline power in M1. The mean of beta frequency was 18.6 Hz (~20 Hz), SD was 2.59 Hz, and the range was 13 to 26 Hz. Notes: CGH = the video of computer graphic hand movements; VRH = the video of real hand movements; M1 = primary motor cortex.

**Figure 3:**
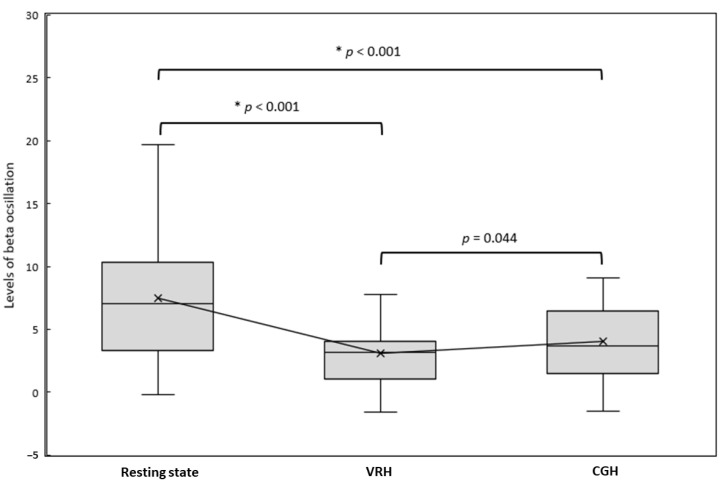
The levels of beta oscillation activities of M1 in the 3 conditions. Based on the Wilcoxon signed-rank test with Bonferroni correction, a *p*-value < 0.017 indicated statistical significance (i.e., 3 comparisons: resting state vs. VRH, resting state vs. CGH, and VRH vs. CGH). An asterisk (*) indicates statistical significance. Notes: CGH = the video of computer graphic hand movements; VRH = the video of real hand movements; M1 = primary motor cortex. Data are presented as median and interquartile range.

Figure 2 illustrates the grand-averaged time–frequency maps and corresponding beta rebound oscillations of M1 under each condition in healthy participants. Based on the Friedman test, there were significant differences in beta oscillatory activities among the 3 conditions (χ^2^(2) = 22.90, *p* < 0.001). In comparison with the resting state condition (median = 7.07, interquartile range (IQR) = 7.04), the beta oscillatory activities were significantly lower in the 2 observation conditions: VRH (median = 3.17, IQR = 3.02; Z = −3.85, *p* < 0.001) and CGH (median = 3.70, IQR = 4.98; Z = –3.73, *p* < 0.001). However, the differences in beta oscillatory activities between the VRH and CGH conditions did not reach statistical significance (Z = −2.02, *p* = 0.044) (Figure 3). Lower beta oscillatory activities indicated greater M1 cortical activations. The results indicated that M1 activations were more pronounced in the VRH and CGH conditions than in the resting state condition. However, the M1 activations in the VRH and CGH conditions were similar.

## 4. Discussion and Conclusions

This study demonstrated that M1 cortical activations during the observation of the video of real hand movements (i.e., VRH condition) and during the observation of the video of computer graphic hand movements (i.e., CGH condition) were similar, whereas M1 activations in the VRH and CGH conditions were significantly different from those in the resting state condition.

Our findings indicated that visual stimuli of movements provided by either the video of a real hand or a computer graphic hand induced M1 activations. These results are similar to the findings of previous studies on body ownership employing the rubber hand illusion paradigm [25,26]. Increased corticospinal excitability and facilitation of the motor system were found in healthy people who observed the actions of another person in AO [26]. Furthermore, as in patients with amputations, corticospinal pathways were inhibited in healthy participants who experienced the illusion of a missing body-part in VR [25]. This study provides evidence that visual stimuli generated by AO and VR may be useful and pragmatic training for motor rehabilitation in clinical settings. 

The visual realism of a computer graphic hand or VR hand might be affected by a triad composed of VR technology, the complexity of observed movements, and participants’ individual perceptual traits. These factors have considerable impacts on brain activations during the observation of computer graphic or VR movements. A previous study reported that greater resemblance of a simulated body part to the actual body part was correlated with a stronger sense of ownership [27]. Due to advances in VR technology, the visual realism of VR-generated presentations has been increased. As a previous study reported, the illusion of VR-generated limbs is similar to that of mirrored limbs; furthermore, the activation in the primary sensorimotor cortex is stronger when VR-generated limbs are observed than when mirrored limbs are [28], which is contrary to our findings. One possible reason for this difference might be that participants are immersed in a VR environment while using a head-mounted display, rather than watching movements presented on a two-dimensional screen. Another explanation might be that the finger flexion and clenching movements were similar to the movements of gripping and releasing a ball, but the latter additionally provided visual clues of gripping a ball (i.e., transitive movements), which may influence the degree of visual realism. 

In addition, individual differences in the perceived vividness of virtual or illusory body parts among the participants may lead to different results [29]. In the study of Perani et al., different degree of realism in real and VR hands, including high (realistic) and low (coarse) VR hands, led to different activation patterns [11]. However, in the study of Brand et al., they found that there was no significant difference in brain activation between virtual (more realistic) hand and shadow (less realistic) hand while observing [30], which was in line with our findings. Thus, between the VR and real context, the much closer physical characteristics of visual stimuli perceived, the much similar effects of brain activations evoked. In our study, given the very similar visual stimuli of CGH condition to that of VRH condition, there was no significant difference between the VRH and CGH conditions, but more participants had lower M1 beta rebound oscillation in the VRH condition than in the CGH condition. The present study reveals that although the visual stimuli are relatively real in the VRH condition, unlike that in the CGH condition, how the participants perceive what they see modulates the visual realism. 

In this study, the stimulus intensity was set as 20% above the motor threshold to induce a brief thumb movement (mean = 3.57 mA). This intensity was painless for the participants and allowed us to obtain cortical responses with a good signal-to-noise ratio. The stimulus intensity was the same within each participant among different experimental conditions. Thus, the cortical differences between different conditions could not be attributed to the peripheral somatosensory inputs. However, the application of intermittent electrical stimuli might distract the participants from the perception of or the attention to the visual stimuli. Further research is warranted to examine the potential interference of electrical stimuli to the visual stimuli.

The key contribution of this study is the finding that observing a video of real-hand movements (i.e., VRH condition) and computer graphic-hand movements (i.e., CGH condition) had comparable effects on M1 cortical activations; they were more reactive than their counterparts in the resting state. In clinical practice, the rehabilitative treatment approaches of observation-based learning and visual stimuli, namely, action observation therapy and virtual reality training, might be used as alternatives to each other. 

Some limitations of this study need to be addressed. First, motor imagery of the imagined hand would decrease beta oscillatory activities in the contralateral sensorimotor cortex [31]. During the VRH and CGH conditions, simultaneously engaging in motor imagery while observing hand movements might affect the M1 beta rebound oscillations. Second, the counterbalancing order did not include the resting state condition in this study. It is further suggested that all the experimental conditions, including resting state condition, have to be counterbalanced across the participants to reduce the order effect. Third, the MEG experimental design of this study narrowed to the investigation of beta rebound in M1, which restricted the comprehensive exploration of the activation and functional connectivity in visual cortex and other areas of the mirror neuron system. Further work is suggested to investigate the activation and functional connectivity in other areas of the mirror neuron system, such as PMv, PMd, SMA, IFG, and IPL. Fourth, the modest scale of the study (20 healthy participants) may have partially contributed to the statistically non-significant differences between the two experimental conditions. Further larger-scale studies to compare the neural mechanisms in patients with neurological conditions (e.g., stroke) and in healthy controls are recommended. 

In conclusion, the observation in a video of hand movements performed by a healthy person’s real hand and of such movements performed by a computer graphic hand evoked comparable motor cortical activations in healthy adults. This study provides a neurophysiological basis for the link between the observation of human movements and production of the same movements, and for the potential use of action observation therapy and virtual reality in rehabilitation practice. Further research is suggested to investigate the neural mechanisms modulated by the different visual presentations of action observation via videos and virtual movements in patients with stroke.

## Data Availability

The data presented in this study are available on request from the corresponding author. The data are not publicly available due to the confidentiality.

## References

[1] Keysers C., Gazzola V. (2010). Social neuroscience: Mirror neurons recorded in humans. Curr. Biol..

[2] Sale P., Ceravolo M.G., Franceschini M. (2014). Action observation therapy in the subacute phase promotes dexterity recovery in right-hemisphere stroke patients. BioMed Res. Int..

[3] Buccino G. (2014). Action observation treatment: A novel tool in neurorehabilitation. Philos. Trans. R Soc. Lond. B Biol. Sci..

[4] Small S.L., Buccino G., Solodkin A. (2012). The mirror neuron system and treatment of stroke. Dev. Psychobiol..

[5] Sale P., Franceschini M. (2012). Action observation and mirror neuron network: A tool for motor stroke rehabilitation. Eur. J. Phys. Rehabil. Med..

[6] Liepert J., Greiner J., Dettmers C. (2014). Motor excitability changes during action observation in stroke patients. J. Rehabil. Med..

[7] Laver K.E., Lange B., George S., Deutsch J.E., Saposnik G., Crotty M. (2018). Virtual reality for stroke rehabilitation. Stroke.

[8] Bagce H.F., Saleh S., Adamovich S.V., Tunik E. (2012). Visuomotor gain distortion alters online motor performance and enhances primary motor cortex excitability in patients with stroke. Neuromodulation.

[9] Saleh S., Adamovich S.V., Tunik E. (2014). Mirrored feedback in chronic stroke: Recruitment and effective connectivity of ipsilesional sensorimotor networks. Neurorehabil. Neural Repair.

[10] Tunik E., Saleh S., Adamovich S.V. (2013). Visuomotor discordance during visually-guided hand movement in virtual reality modulates sensorimotor cortical activity in healthy and hemiparetic subjects. IEEE Trans. Neural Syst. Rehabil. Eng..

[11] Perani D., Fazio F., Borghese N.A., Tettamanti M., Ferrari S., Decety J., Gilardi M.C. (2001). Different Brain Correlates for Watching Real and Virtual Hand Actions. NeuroImage.

[12] Marshall P.J., Meltzoff A.N. (2011). Neural mirroring systems: Exploring the EEG μ rhythm in human infancy. Dev. Cogn. Neurosci..

[13] Meltzoff A.N., Marshall P.J. (2018). Human infant imitation as a social survival circuit. Curr. Opin. Behav. Sci..

[14] Meltzoff A.N., Ramírez R.R., Saby J.N., Larson E., Taulu S., Marshall P.J. (2018). Infant brain responses to felt and observed touch of hands and feet: An MEG study. Dev. Sci..

[15] Cheng C.H., Sun H.H., Weng J.Q., Tseng Y.J. (2017). Differential motor cortex excitability during observation of normal and abnormal goal-directed movement patterns. Neurosci. Res..

[16] Zhu J.D., Cheng C.H., Tseng Y.J., Chou C.C., Chen C.C., Hsieh Y.W., Liao Y.H. (2019). Modulation of Motor Cortical Activities by Action Observation and Execution in Patients with Stroke: An MEG Study. Neural Plast..

[17] Wilson T.W., Slason E., Asherin R., Kronberg E., Reite M.L., Teale P.D., Rojas D.C. (2010). An extended motor network generates beta and gamma oscillatory perturbations during development. Brain Cogn..

[18] Hari R., Salmelin R. (1997). Human cortical oscillations: A neuromagnetic view through the skull. Trends Neurosci..

[19] Pfurtscheller G., Lopes da Silva F.H. (1999). Event-related EEG/MEG synchronization and desynchronization: Basic principles. Clin. Neurophysiol..

[20] Hari R., Forss N., Avikainen S., Kirveskari E., Salenius S., Rizzolatti G. (1998). Activation of human primary motor cortex during action observation: A neuromagnetic study. Proc. Natl. Acad. Sci. USA.

[21] Taulu S., Simola J. (2006). Spatiotemporal signal space separation method for rejecting nearby interference in MEG measurements. Phys. Med. Biol..

[22] Tadel F., Baillet S., Mosher J.C., Pantazis D., Leahy R.M. (2011). Brainstorm: A user-friendly application for MEG/EEG analysis. Comput. Intell. Neurosci..

[23] Huang M.X., Mosher J.C., Leahy R.M. (1999). A sensor-weighted overlapping-sphere head model and exhaustive head model comparison for MEG. Phys. Med. Biol..

[24] Cheng C.H., Tseng Y.J., Chen R.S., Lin Y.Y. (2016). Reduced functional connectivity of somatosensory network in writer’s cramp patients. Brain Behav..

[25] Kilteni K., Grau-Sanchez J., De Las Heras M.V., Rodriguez-Fornells A., Slater M. (2016). Decreased Corticospinal Excitability after the Illusion of Missing Part of the Arm. Front. Hum. Neurosci..

[26] Schutz-Bosbach S., Mancini B., Aglioti S.M., Haggard P. (2006). Self and other in the human motor system. Curr. Biol..

[27] Tsakiris M., Carpenter L., James D., Fotopoulou A. (2010). Hands only illusion: Multisensory integration elicits sense of ownership for body parts but not for non-corporeal objects. Exp. Brain Res..

[28] Diers M., Kamping S., Kirsch P., Rance M., Bekrater-Bodmann R., Foell J., Trojan J., Fuchs X., Bach F., Maaß H. (2015). Illusion-related brain activations: A new virtual reality mirror box system for use during functional magnetic resonance imaging. Brain Res..

[29] Bekrater-Bodmann R., Foell J., Diers M., Flor H. (2012). The perceptual and neuronal stability of the rubber hand illusion across contexts and over time. Brain Res..

[30] Brand J., Piccirelli M., Hepp-Reymond M.-C., Eng K., Michels L. (2020). Brain Activation During Visually Guided Finger Movements. Front. Hum. Neurosci..

[31] Brinkman L., Stolk A., Dijkerman H.C., de Lange F.P., Toni I. (2014). Distinct roles for alpha- and beta-band oscillations during mental simulation of goal-directed actions. J. Neurosci..

